# Acquisition of a single grid-based phase-contrast X-ray image using instantaneous frequency and noise filtering

**DOI:** 10.1186/s12938-022-01061-z

**Published:** 2022-12-27

**Authors:** Jae-Suk Yang, Sun-Young Jeon, Jang-Hwan Choi

**Affiliations:** Division of Mechanical and Biomedical Engineering, Graduate Program in System Health Science and Engineering, Ewha Womans University, Seoul, 03760 Republic of Korea

**Keywords:** Phase-contrast X-ray image, Instantaneous frequency, Noise filtering, Fourier analysis, Moire artifact

## Abstract

**Background:**

To obtain phase-contrast X-ray images, single-grid imaging systems are effective, but Moire artifacts remain a significant issue. The solution for removing Moire artifacts from an image is grid rotation, which can distinguish between these artifacts and sample information within the Fourier space. However, the mechanical movement of grid rotation is slower than the real-time change in Moire artifacts. Thus, Moire artifacts generated during real-time imaging cannot be removed using grid rotation. To overcome this problem, we propose an effective method to obtain phase-contrast X-ray images using instantaneous frequency and noise filtering.

**Result:**

The proposed phase-contrast X-ray image using instantaneous frequency and noise filtering effectively suppressed noise with Moire patterns. The proposed method also preserved the clear edge of the inner and outer boundaries and internal anatomical information from the biological sample, outperforming conventional Fourier analysis-based methods, including absorption, scattering, and phase-contrast X-ray images. In particular, when comparing the phase information for the proposed method with the x-axis gradient image from the absorption image, the proposed method correctly distinguished two different types of soft tissue and the detailed information, while the latter method did not.

**Conclusion:**

This study successfully achieved a significant improvement in image quality for phase-contrast X-ray images using instantaneous frequency and noise filtering. This study can provide a foundation for real-time bio-imaging research using three-dimensional computed tomography.

## Background

X-rays have been employed for numerous applications in a wide range of fields, including clinical diagnoses, product inspection, material analysis, and airport security. However, traditional absorption images produced using conventional X-ray techniques only reveal the internal structure of soft tissue (low-Z material) due to the weak absorption contrast caused by the thickness and absorption coefficients of the samples [1, 2]. In contrast, compared to absorption images, scattering and phase-contrast X-ray images can reveal a greater range of internal information for soft tissue based on phase changes. Particularly, scattering images provide scattered signals using micro-structures [3–6], and phase-contrast X-ray images can clearly reveal the phase contrast of the boundaries of internal structures using refractive indices [7–11].

Phase-contrast X-ray images have been employed to reveal internal information for medical and biological samples in various ways. For example, they can be used to observe tumor masses due to the relatively slow variation in the integrated phase shift [12]. However, phase-contrast X-ray images obtained using computed tomography (CT) can accurately reveal the structures of different types of soft tissue in coronary artery autopsy samples to understand heart disease and visualize microscopic structures, such as lipid-rich plaques, individual adipose cells, ensembles of foam cells, and thin fibrous caps [13]. Similar to confocal microscopy, CT has also been used to observe the muscular structures of unstained whole zebrafish at a high sub-cellular resolution [14]. Although the scattered signals produced by microbubble contrast agents negatively affect the internal information obtained from CT images, CT has been employed to accurately visualize the 3D architecture of murine cardiovascular vessels [15], while dark-field scattering CT images have also successfully detected cracks inside teeth to diagnose the causes of tooth pain [16]. In addition, CT image patterns can be used to examine the condition of thin cartilage attached to the bones of patients and provide important clues for diagnosing inflammation and pain associated with cartilage [17].

Although there are various techniques for obtaining phase-contrast X-ray images, grating-based imaging has recently emerged as a promising method due to its ability to simultaneously generate absorption, scattering, and differential phase-contrast X-ray images. Particularly, this technique can generate scattering and phase-contrast X-ray images by converting the incoherence of conventional X-ray sources into coherence using a grating process [18–21]. However, excessive time requirements and mechanical errors caused by grid motion during the phase-shift process [22, 23] have limited the practical application of grating-based imaging for real-time bio-imaging. Additionally, the alignment of the grids used in the experimental setup for this technique must be precise to achieve the desired imaging conditions [24, 25], and this tedious alignment process has restricted the widespread adoption of this technique.

To overcome these problems, Wen et al. proposed a single-grid phase-contrast X-ray imaging system to obtain absorption, scattering, and phase-contrast X-ray images using Fourier analysis [26, 27]. However, the presence of Moire artifacts in single-grid phase-contrast X-ray images, which are caused by the grid, remains a serious limitation. To remove these artifacts, a previous study [28] proposed the use of grid rotation on a grid plane to distinguish the spectral peaks between the sample and the artifacts in the Fourier domain. Consequently, this enables the extraction of the artifacts from the phase-contrast X-ray image. However, noise filtering through software processing and the mechanical movement of grid rotation affect real-time measurement. Due to current levels of computing power, noise filtering can be performed immediately after or during image acquisition. Furthermore, previous studies have proposed the use of non-iterative integration and sparse domain regularized stripe decomposition without mechanical movement for noise filtering to remove the streak and ring artifacts during the CT process [29, 30]. However, the response of grid rotation to the noise (e.g., Moire artifacts) generated during image acquisition is slow because the mechanical movement of grid rotation is generally slower than the image acquisition speed, and grid rotation cannot modify the image after it is acquired. Therefore, a noise-filtering method that can be employed for real-time measurements is required.

To overcome the mechanical limitations of grid rotation, in this study, we proposed the acquisition of phase-contrast X-ray images using instantaneous frequency and noise filtering. During the acquisition process, an original phase-contrast X-ray image containing sample information with noise was acquired using instantaneous frequency. Subsequently, noise filtering was employed to remove the noise in the image. Instantaneous frequency and noise filtering thus enabled the acquisition of a filtered phase-contrast X-ray image from a single raw image obtained from a detector without using grid rotation, which means that the method proposed in this study has the potential to be used in real-time bio-imaging. In addition, the proposed noise-filtering process exhibited outstanding performance in terms of simultaneously removing noise while preserving detailed information from inside the sample tissue. Compared to absorption x-axis gradient images, the images obtained using the proposed method were able to clearly distinguish between different types of tissue and provide detailed information from inside the bone and soft tissue of the sample.

## Result

### Qualitative image analysis using bio-samples

**Fig. 1:**
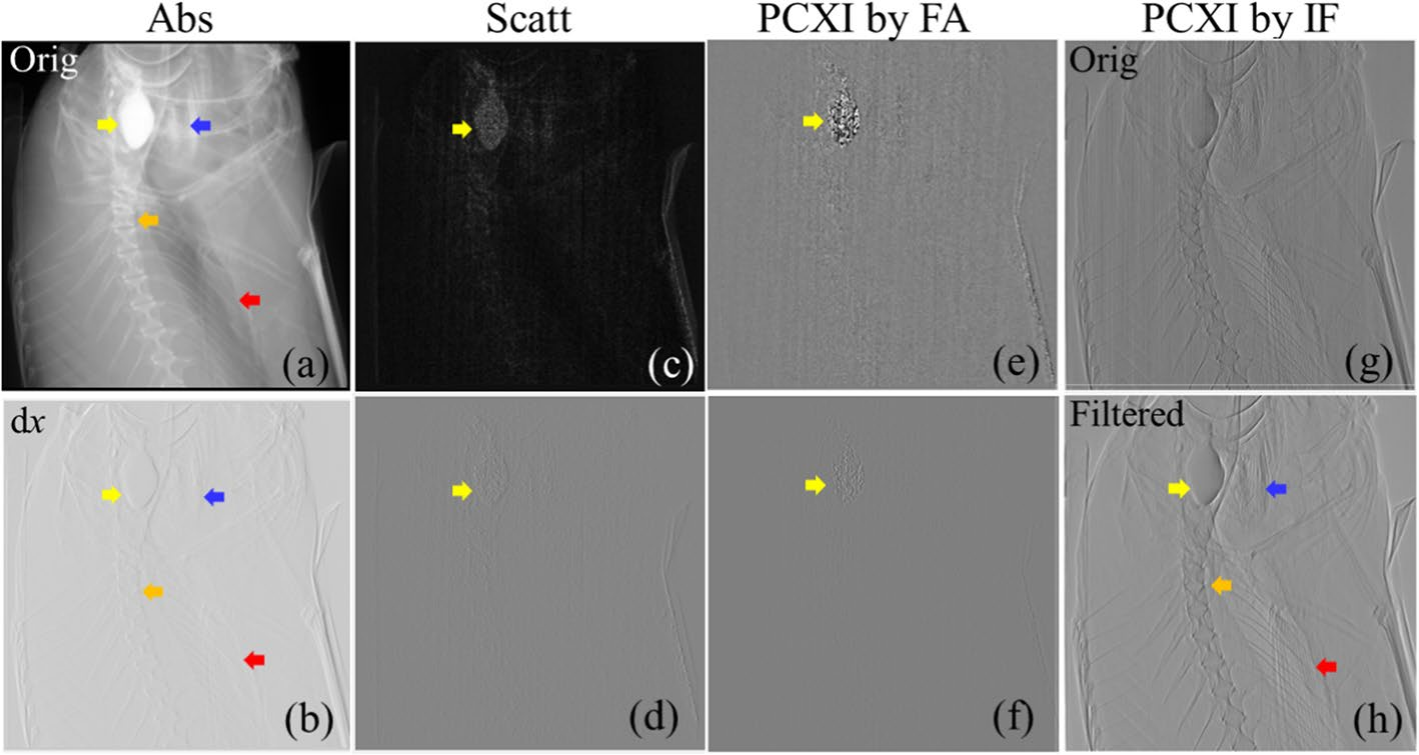
**a**–**h** Eight subset images of the head of the bio-sample. FA: Fourier analysis; IF: instantaneous frequency; Abs: absorption image; Scatt: scattering image; PCXI: phase-contrast X-ray image; dx: x-axis gradient image; Orig: original

Figure 1 presents the images acquired for the head of the fish used as the bio-sample. Figure 1a, c, e represents the absorption, scattering, and phase-contrast X-ray images of the bio-sample acquired using Fourier analysis, and Fig. 1b, d, f represents their corresponding x-axis gradient images, respectively. Figure 1g, h shows the original phase-contrast X-ray image of the bio-sample acquired using instantaneous frequency and its corresponding filtered image obtained using the proposed method, respectively. The information of the bone and soft tissues of the bio-sample, including the gill, fin, and vertebral tissues (red, orange, and blue arrows, respectively) are presented in Fig. 1a. Figure 1c, e shows an image of the soft tissue (indicated by the yellow arrow) with the noise pattern. The tissue boundary in Fig. 1b is less clear compared to that in Fig. 1a. The information from the bio-sample is more ambiguous in Fig. 1d, f than in Fig. 1b, while Fig. 1h has the best image quality. It makes a clear distinction between the soft tissue (yellow arrow) and the surrounding tissue under operating conditions of 22 kVp, and detailed information on the gill, fin, and vertebral tissue (red, orange, and blue arrows, respectively) can be observed. Figure 1g also shows the internal information from the bio-sample; however, noise is also present in the image.

**Fig. 2:**
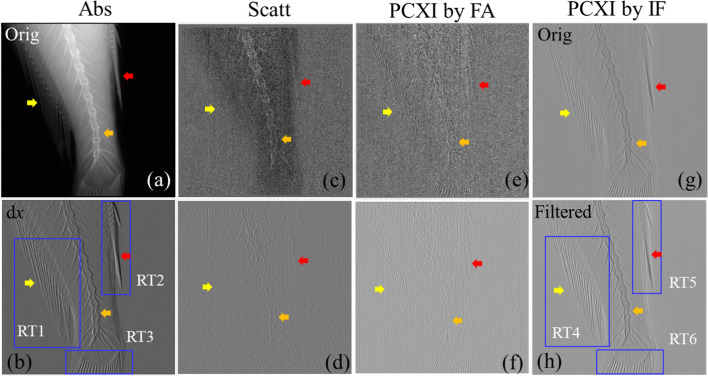
**a**–**h** Eight subset images of the tail of the bio-sample. FA: Fourier analysis; IF: instantaneous frequency; Abs: absorption image; Scatt: scattering image; PCXI: phase-contrast X-ray image; dx: x-axis gradient image; Orig: original; RT: rectangle

Figure 2 presents images for the tail of the bio-sample. Figure 2a, c, e displays the absorption, scattering, and phase-contrast X-ray images acquired using Fourier analysis, while Fig. 2b, d, f represents their corresponding x-axis gradient images. Figure 2g, h shows the original phase-contrast X-ray image obtained using instantaneous frequency and the image obtained using the proposed method, respectively. Figure 2a presents the bone and fin structures (red, orange, and yellow arrows) of the bio-sample. Figure 2c, e shows the bone and outer boundary between the fish air, but severe noise can be observed. The image of the boundary in Fig. 2b is clearer than that in Fig. 2a, while the internal structure visible in Fig. 2d, f is more ambiguous than Fig. 2b. Overall, Fig. 2h provides the clearest information on the various internal structures, with the fins and vertebral tissue (red, orange, and yellow arrows) more clearly expressed than those in Figs. 2a–f. Figure 2g presents the internal information from the bio-sample with periodic noise.

### Comparison of absorption gradient and proposed phase-contrast images using instantaneous frequency

**Fig. 3:**
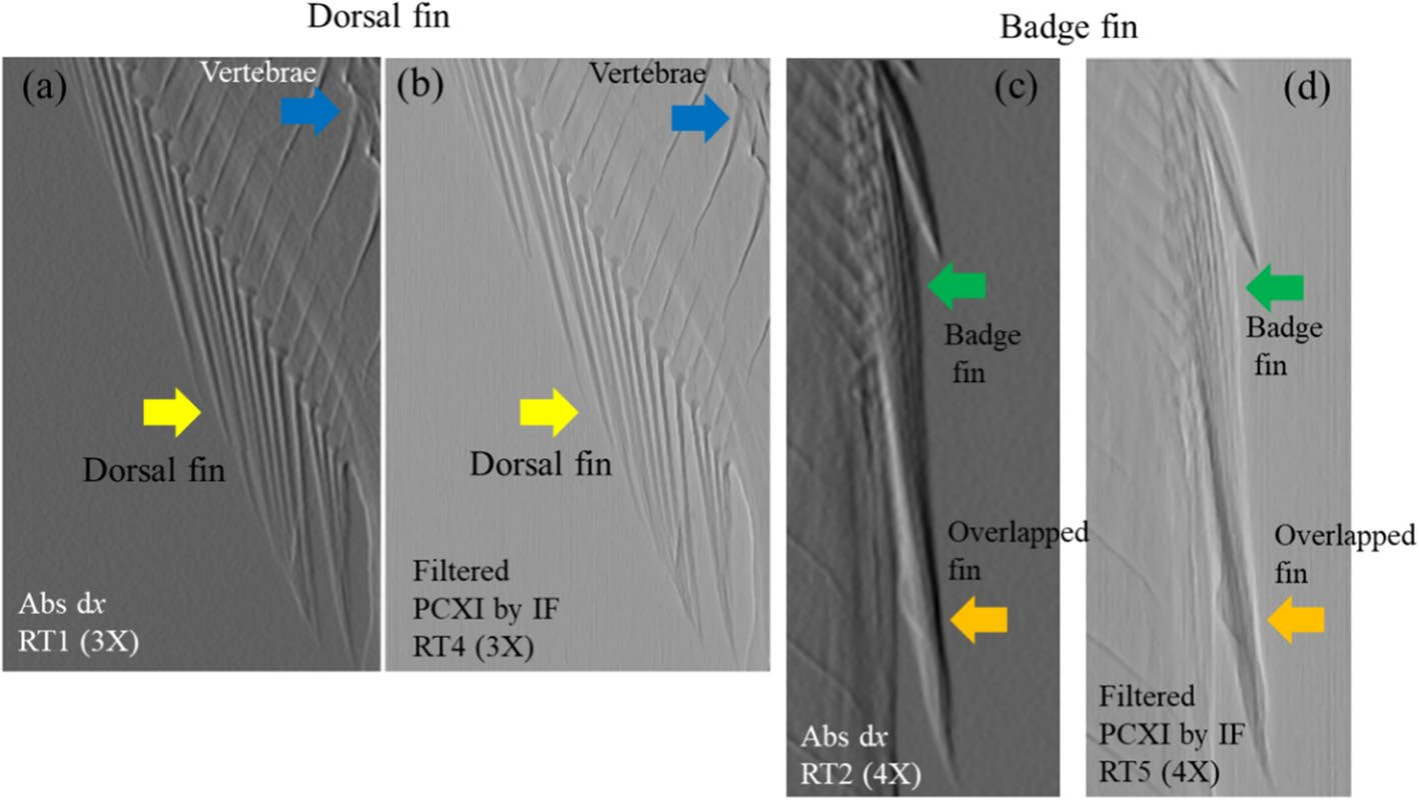
Comparison of the areas indicated by the blue boxes in Fig. 2 (RT1, RT2, RT4, and RT5: dorsal and badge fin) for the **a**, **c** absorption *x-axis* gradient image and **b**, **d** phase-contrast X-ray image obtained using instantaneous frequency. IF: instantaneous frequency; Abs: absorption image; PCXI: phase-contrast X-ray image; dx: *x-axis* gradient image; RT: rectangle

To verify the performance of the proposed imaging method, the absorption x-axis gradient image in Fig. 2b was selected as a reference image and compared to the proposed filtered phase-contrast X-ray image in Fig. 2h because this image exhibited the best quality of those acquired using Fourier analysis. Figure 3a, c presents a magnification of the blue boxes RT1 and RT2, respectively; Figs. 2b and 3b, d present a magnification of the blue boxes RT4 and RT5, respectively, in Fig. 2h. Figure 3a, b shows the elongated fine patterns of the dorsal fin (yellow arrow). The boundary of the dense fin structures in Fig. 3b is clearer than that in Fig. 3a. In addition, a sharp change from white to black in each fin structure can be observed in Fig. 3a. Furthermore, compared to Fig. 3c, more delicate structures of the badge fin (green arrow) are visible in Fig. 3d, and the internal information on the overlapping fin structures (orange arrow) is more clearly expressed. Additionally, compared to Fig. 3a, the vertebrae of the fish are more clearly visible in Fig. 3b (blue arrow).

### Noise removal using an adaptive notch and multi-resolution structurally varying bitonic filters

**Fig. 4:**
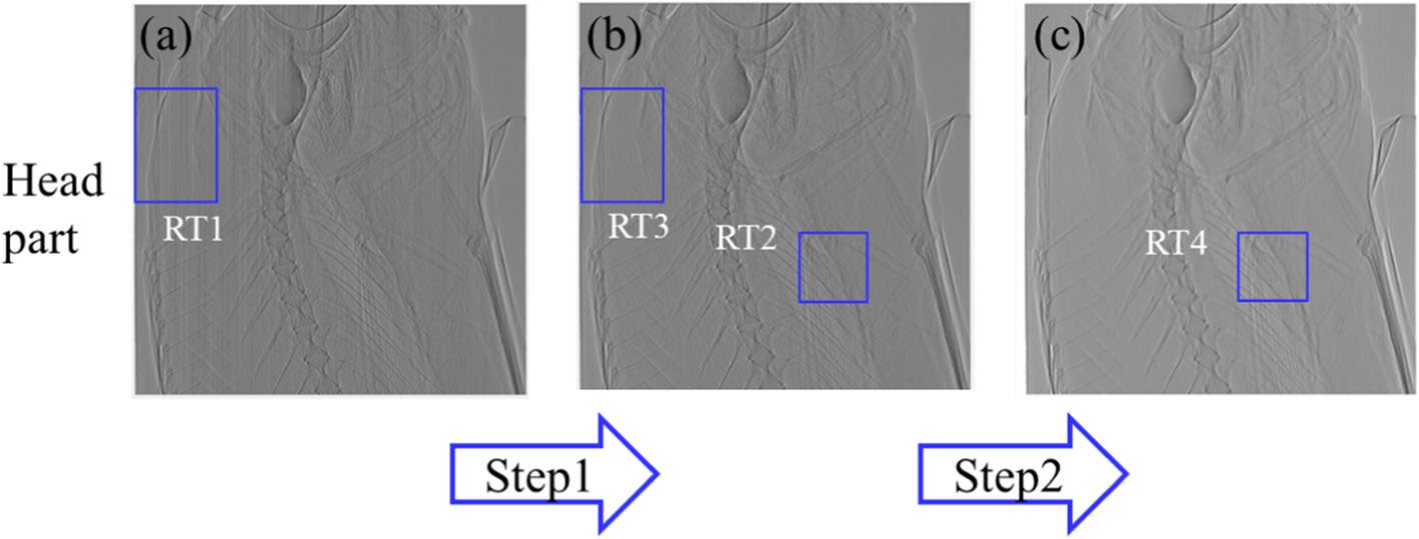
Images showing the noise-filtering process: adaptive Gaussian notch (Step 1: **a** to **b**) and multi-resolution structurally varying bitonic filter (Step 2: **b** to **c**). RT: rectangle

**Fig. 5:**
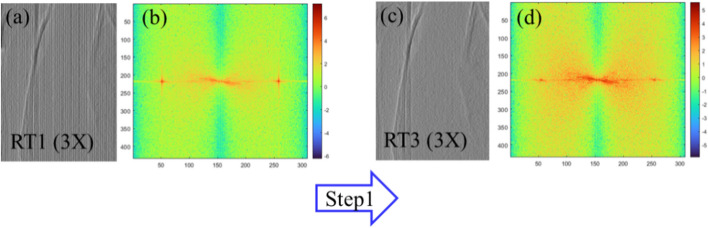
Image filtering of the areas indicated by the blue boxes in Fig. 4**a**, **c** (i.e., RT1 and 3) using an adaptive Gaussian notch filter (Step 1: **a** to **c**) and the corresponding spectral images. **b**, **d** The spectral images of **a** and **c**, respectively. RT: rectangle

Figure 4 presents the results of the noise filtering conducted in two steps: (1) the use of an adaptive Gaussian notch filter and (2) the use of a multi-resolution structurally varying bitonic filter [31]. The multi-resolution structurally varying bitonic filter was selected from among three types of bitonic filter (Matlab code: mvbitonic2 with a parameter value of 7). To identify the effect of the adaptive Gaussian notch filter (Step 1), a filtered image was obtained (Fig. 5c). Compared to the image in Fig. 5a, the periodic noise in the filtered image was significantly lower. The spectral image of Fig. 5a is presented as Fig. 5b, while the spectral image of Fig. 5c is presented as Fig. 5d. Using these spectral images, we evaluated the effect of the notch filter. Compared with Fig. 5b, the vertical and horizontal outer components of the two crosses in Fig. 5d were significantly suppressed. In signal processing, a band stop filter is used to block the spatial frequency range of unwanted noise. In this context, a notch filter has a narrow spatial frequency range, which is also referred to as the notch frequency range [32]. To identify the effect of the multi-resolution structurally varying bitonic filter (Step 2), we conducted noise filtering with a parameter value of 7. The filter converted the image in Fig. 8a into the image in the bottom row, fourth column in Fig. 8b.

## Discussion

The proposed method introduced in this study produces excellent image quality. In particular, the distinction between the target soft tissue and surrounding tissue is clear (see "Soft tissue segmentation ability of the proposed method" section). The proposed images present fine structures and a clear distinction between bone and soft tissue in the fin compared to the absorption *x-axis* gradient images (see "Difference between absorption *x-axis* gradient and phase-contrast X-ray images" section). During noise filtering, the proposed images are able to preserve detailed information while removing noise (see "Two-step noise filtering in the phase-contrast X-ray image" section).

### Relation between noise, X-ray source energy, and noise filtering

Figures 1 and 2 summarize the relationship between energy and noise in absorption, scattering, and phase-contrast X-ray images. It is generally well known that low-energy images show better image quality than high-energy images. For example, Gromann et al. [33] reported that phase-contrast X-ray images and scattering low-energy (30 kVp) images reveal clearer and more detailed information on the soft tissue in 4-cm-thick breast samples than do high-energy images (40 kVp). However, some studies have reported contrasting findings. A previous study [34] reported that the internal information from a sample is not visible in low-energy images, which can be attributed to the fact that the scattering signals caused by the micro-structures in low-energy images are more prominent than that in high-energy images. Consequently, the scattering signals in phase-contrast X-ray images at a low energy result in noise, which can lead to the loss of detailed information from a sample. In addition, compared to high-energy (90 and 70 kVp) images, the scattering signals for low-energy (50 kVp) images obscure details of the internal structure of a sample. In this study, we avoided the problems associated with scattering signals in low-energy images using noise filtering.

### Soft tissue segmentation ability of the proposed method

Compared to the yellow arrows in Fig. 1a, c, e, the yellow arrow in Fig. 1h at 22 kVp indicates a clear distinction between two different types of soft tissue. This demonstrates that the proposed images have the potential for use in medical applications because they have the segmentation ability required to distinguish the target tissue, such as cancer cells, biopsied lesions, fat tissue, collagen strands, glandular tissue, or macro-or micro-calcification, from the surrounding soft tissue. Previous studies have reported that this method can detect tissues of interest. Zhao et al. [35] demonstrated that palpable tumors or biopsied lesions that cannot be detected using mammograms can be detected using phase-contrast X-ray images (60 keV). In their study, the segmentation of an isolated tumor from the surrounding tissue (e.g., skin, lobules, and lactiferous ducts) was possible using phase-contrast X-ray images, with the 3D volume of the tumor estimated to be approximately 2.7 cm^3^. Diemoz et al. [36] acquired a phase-contrast X-ray image (60 keV) that revealed the various sub-tissues (fat tissue, collagen strands, glandular tissue, and macro-calcification) in a breast cancer tissue sample from a patient. Baran et al. [37] also reported propagation-based phase-contrast mammographic tomography data for cancer in a breast tissue sample from a patient. The data provided the detailed structures for cancer sub-tissues (e.g., fibrous stromal septa, blood vessels, and benign cysts).

### Difference between absorption *x*-axis gradient and phase-contrast X-ray images

**Fig. 6:**
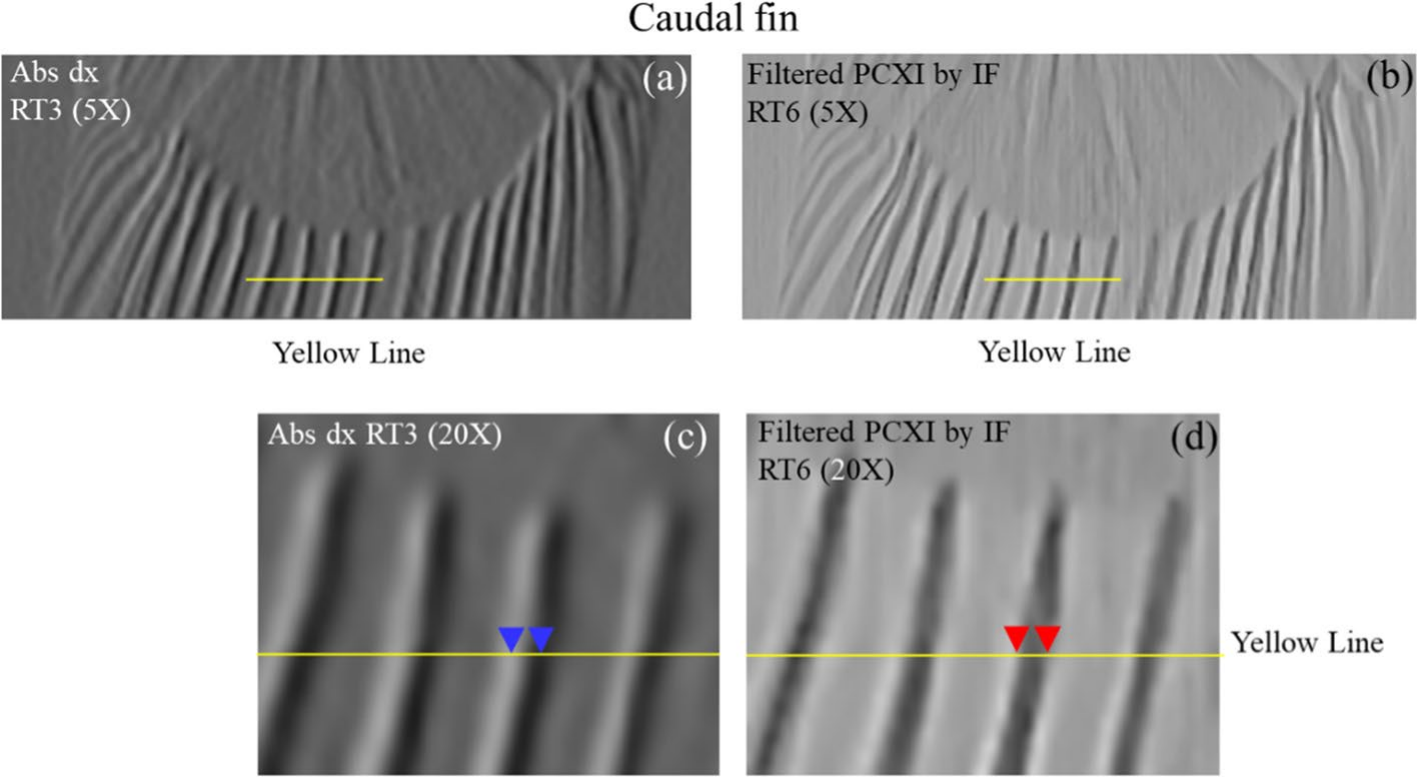
Comparison of the magnified areas indicated by the blue boxes in Fig. 2 (RT3 and 6: caudal fin) for **a**, **c** absorption *x*-axis gradient and **b**, **d** phase-contrast X-ray images. Abs: absorption image; PCXI: phase-contrast X-ray image; IF: instantaneous frequency; dx: *x*-axis gradient image; RT: rectangle

**Fig. 7:**
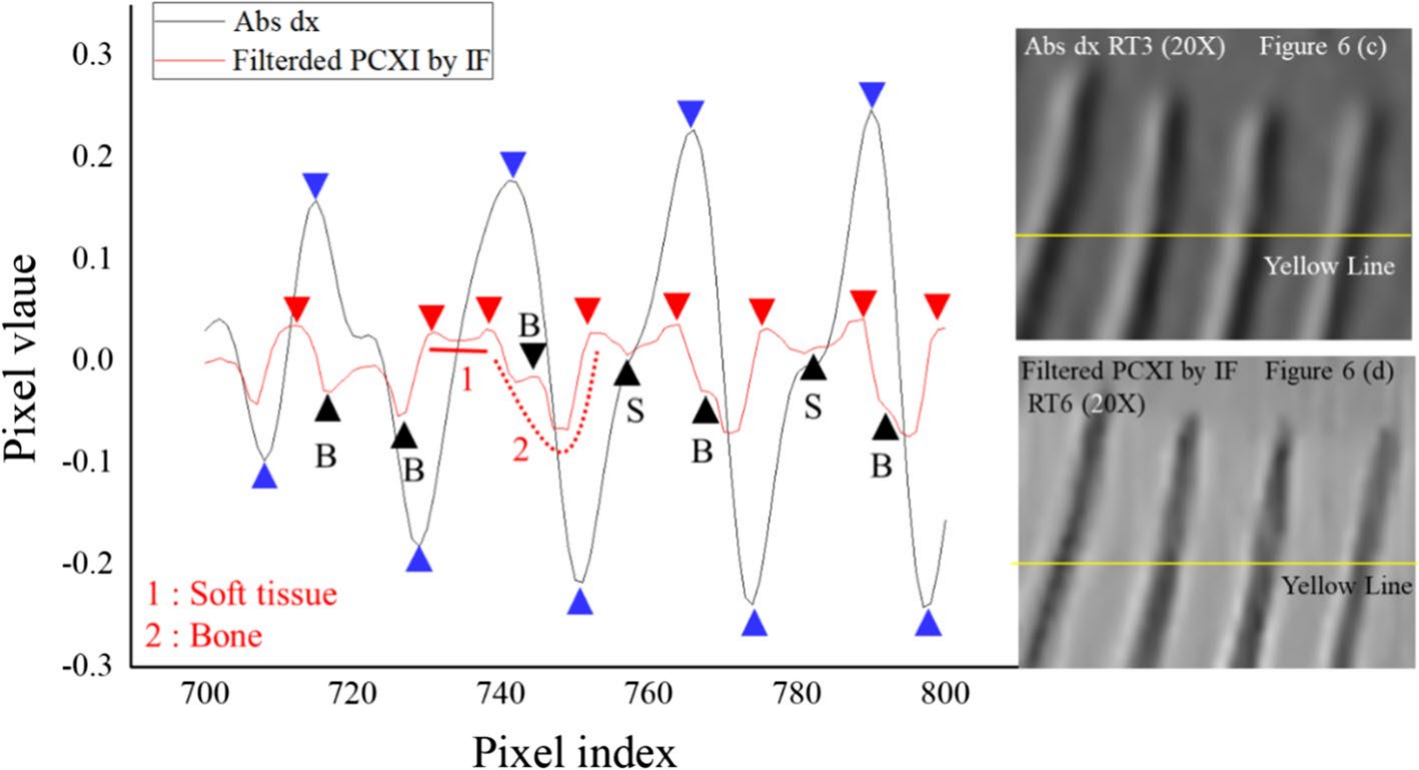
Pixel profiles along the yellow line for the absorption x-axis gradient image in Fig. 6 (c) and the filtered phase-contrast X-ray image in Fig. 6 (d). Abs: absorption image; PCXI: phase-contrast X-ray image; IF: instantaneous frequency; dx: x-axis gradient image; S: soft tissue; B: bone. The solid red line at 1 is constant and the red dotted line at 2 is a quadratic curve

Figures 3, 6, and 7 illustrate the difference between absorption *x-*axis gradient and phase-contrast X-ray images. The details from the images in Fig. 3 were utilized to highlight the features of the proposed and absorption *x-*axis gradient images. The proposed image provided a clear distinction of the outer boundary of the bio-sample in contact with air, a clear view of the inner boundaries of the internal structures, and good preservation of the fine internal structures of the fish. In contrast, the absorption *x*-axis gradient image included a black-to-white change in all structures and a soft and smooth representation of the boundaries of the structures.

Several previous studies have compared absorption *x*-axis gradient and phase-contrast X-ray images using bio-samples. For example, Krejci et al. [38] obtained a clear image of the internal structure of a mouse leg and the dry bone of the head of a hornet, while Du et al. [39] identified the vein tissues in a leaf, which were similar to the vasculature, and Sato et al. [40] extracted the cartilage from the bone of a chicken; however, the method could not identify the inner structure of a tomato, which contains water. Pfeiffer et al. [41] also revealed the delicate structure of the wing of a bird. Generally, differentiating an absorption *x*-axis gradient image increases the noise in the image. To prevent this, Hahn et al. [42] employed a Gaussian-derivative filter with a Gaussian width of 1/2π and found that it mitigated the loss of sample information and improved the image quality. However, they did not accurately compare absorption x-axis gradient and phase-contrast X-ray images. Based on the findings of these studies, we selected a suitable noise filter for our proposed method. The parameters of the noise-filtering technique employed in this study are presented in "Performance evaluation of multi-resolution structurally varying bitonic filter" section.

Figure 6a, c shows the absorption *x*-axis gradient image and Fig. 6b, d shows the proposed phase-contrast X-ray image of the bone and soft tissue in the caudal fin of the bio-sample, and the differences in the pixel profiles of the proposed phase-contrast X-ray and absorption *x*-axis gradient images are clearly presented in Fig. 7. Figure 6a, b presents the 6 × magnified images of the areas in the blue boxes in Fig. 2b, h, while Fig. 6 c, d shows the 20 × magnified area indicated by the yellow line in Fig. 6a, b. The image in Fig. 6d has a clearer division of the boundaries (red triangles) than does the image in Fig. 6 (c) (blue triangles), which suffers from blurred boundaries and a change from white to black for the bone structure.

Figure 7 presents the pixel profiles (row: 1550, col: 700–800) indicated by the yellow lines in Figs. 6 (c) and (d) for the absorption x-axis gradient image and the proposed phase-contrast X-ray image, respectively. The absorption x-axis gradient image was characterized by a smooth curve and a periodic signal with very large differences between the local maxima and minima (indicated by the blue triangles). In contrast, the pixel profile of the proposed image was characterized by more fine-scale changes in the profile (black triangles) and two general patterns: the first type was generally constant (solid red line) and the other was a quadratic curve (red dotted line). In addition, points that separated these two patterns were observed (red triangles).

Based on these pixel profiles, the disadvantages of absorption x-axis gradient images are readily apparent. First, the minima and maxima (blue triangles) hindered the distinction between bone and soft tissue. Secondly, the abrupt change in the pixel values between two points could easily be misunderstood as the boundary between bone and soft tissue. In contrast, the proposed image clearly presented a constant pixel profile (solid red line) that was representative of soft tissue, whereas the quadratic curve (red dotted line) corresponded to bone. Thus, the points between these two patterns (red triangles) corresponded to the boundary between bone and soft tissue. The proposed image also provided detailed information (black triangles) on the soft tissue (S) and bone (B).

### Two-step noise filtering in the phase-contrast X-ray image

Previous studies [43, 44] have provided detailed internal information for a sample through pixel profile analysis. However, these studies did not report the removal of noise from the images. For example, Kneip et al. [45] obtained pixel profiles of a phase-contrast X-ray image and absorption image of the leg of a damselfly. They found that the profile of the phase-contrast X-ray image provided more detailed information, but it contained noise and artifacts. Another study [42] reported that the distinction between noise and the detailed structure of tissue in absorption and phase-contrast X-ray images is ambiguous. To address these problems, we employed a two-step noise-filtering process (Steps 1 and 2) to simultaneously preserve the detailed internal information and reduce noise. This would enable our proposed method to be more suitable for use in medical diagnosis than these past studies.

To remove the residual noise after noise filtering (Step 1), we propose the use of a multi-resolution structurally varying bitonic filter. Figure 5c shows that the filter removed most of the periodic noise from the image, but salt-and-pepper noise could still be observed. Generally, median and average filters are effectively used to filter this type of noise. However, the use of these filters can result in the severe loss of detailed information from the sample. Developing a solution that can satisfy both noise removal and the preservation of detailed information has remained a challenge. To overcome this problem, we proposed a second step to satisfy the two requirements that used the parameter value of three bitonic filters (simple, structurally varying, and multi-resolution structurally varying filters) to vary the quality of the filtered images. In this way, the optimal trade-off between retaining detailed information and removing noise could be determined.

**Fig. 8:**
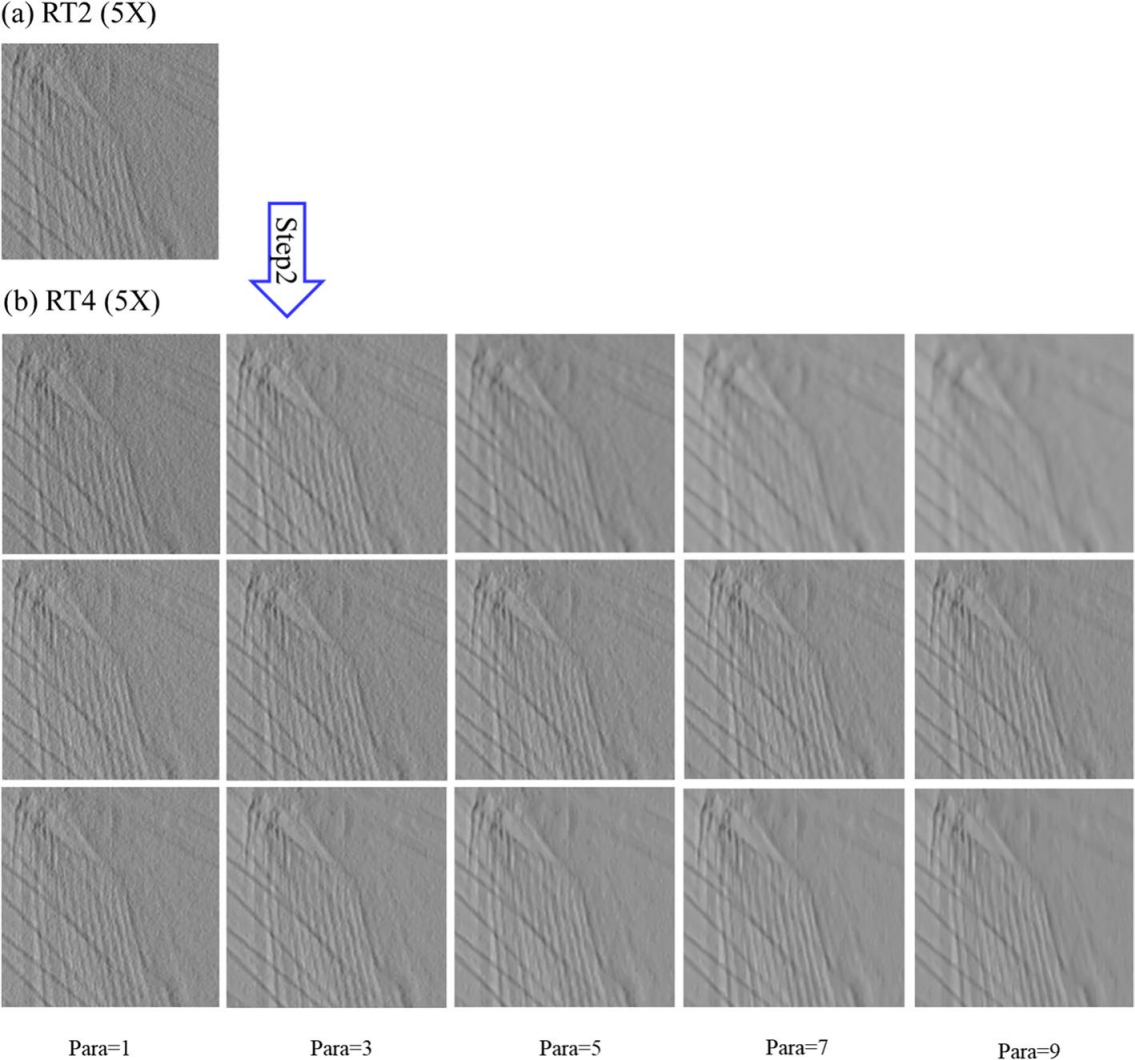
Fifteen filtered images of the areas indicated by the blue boxes in Fig. 4b, c using three bitonic filters with different parameter values (Step 2: Fig. 4 RT2 to RT4). Top row: simple bitonic filter; middle row: structurally varying bitonic filter; and bottom row: multi-resolution structurally varying bitonic filter. RT, rectangle; Para, parameter

Figure 8 presents the images produced with a change in the parameter value (1, 3, 5, 7, and 9) using the three filters. The diameter of the mask was used as the parameter value in the simple bitonic filter, while the size (2 × parameter + 1) of the region containing various masks was used as the parameter value for the structurally varying bitonic and multi-resolution structurally varying bitonic filters. The mask was the coverage area of the three filters. Figure 8b shows that the three bitonic filters removed noise from the image. However, an increase in the blurring effect with a change in the parameter value (≥ 5) was observed in the images filtered using the simple bitonic filter. In contrast, the structurally varying bitonic and multi-resolution structurally varying bitonic filters provided more detailed and clear information for a parameter value ≥ 5. In particular, the images obtained using the structurally varying bitonic filter exhibited detailed information, but this information could not be clearly observed because it was obscured by unremoved residual noise. In contrast, the multi-resolution structurally varying bitonic filter removed most of the residual noise from the images, and the detailed information was generally well preserved. In addition, the analysis of the six images obtained using the multi-resolution structurally varying bitonic and structurally varying bitonic filters at a parameter value of ≥ 5 in Fig. 8b revealed that the multi-resolution structurally varying bitonic filter was more effective for the reduction of residual noise. Based on these results, we selected a parameter value of 7 for the multi-resolution structurally varying bitonic filter as the optimal conditions.

### Performance evaluation of multi-resolution structurally varying bitonic filter

To quantitatively evaluate the noise removal performance and the preservation of detailed information using the multi-resolution structurally varying bitonic filter, the peak signal-to-noise ratio (PSNR), mean squared error (MSE), and structural similarity index map (SSIM) values were calculated for the reference and comparison images over pixel profile for the 15 images in Fig. 8b. The image in the bottom row and 5th column (multi-resolution structurally varying bitonic filter with a parameter value of 9) in Fig. 8b was used as the reference image to calculate the three values because it was generally clearer than the other images. The PSNR, MSE, and SSIM values are listed in Table 1. The optimal conditions for all three metrics were obtained for the image obtained under the selected conditions of the proposed filter (multi-resolution structurally varying bitonic filter with a parameter value of 7). This indicates that the selected conditions for the proposed filter led to remarkable noise removal performance.

**Table 1:** Peak signal-to-noise ratio (PSNR), mean squared error (MSE), and structural similarity index map (SSIM) values for the reference and comparison images

Image quality	Reference:	RT4 (5X) Bottom row, 5st column image					
	Comparison:	RT4 (5X)					RT2 (5X)
		1st column image	2st	3st	4st	5st	
PSNR value (dB)	Top row	12.42	19.89	20.1	17.52	16.15	10.04
	Middle row	12.95	15.25	17.8	20.42	22.74	
	Bottom row	14.71	18.91	22.7	27.54 ^1^		
MSE value	Top row	0.011	0.0047	0.0046	0.0062	0.0072	0.015
	Middle row	0.01	0.008	0.006	0.0044	0.0034	
	Bottom row	0.0085	0.0052	0.0034	0.0019 ^1^		
SSIM value	Top row	0.86	0.95	0.96	0.92	0.89	0.79
	Middle row	0.87	0.91	0.94	0.96	0.98	
	Bottom row	0.91	0.96	0.98	0.99 ^1^		

Reference image: bottom row, fifth column of RT4. Comparison image: the 14 images of RT4 excluding the reference image and the one image of RT2 in Fig. 8. RT: rectangle

**Fig. 9:**
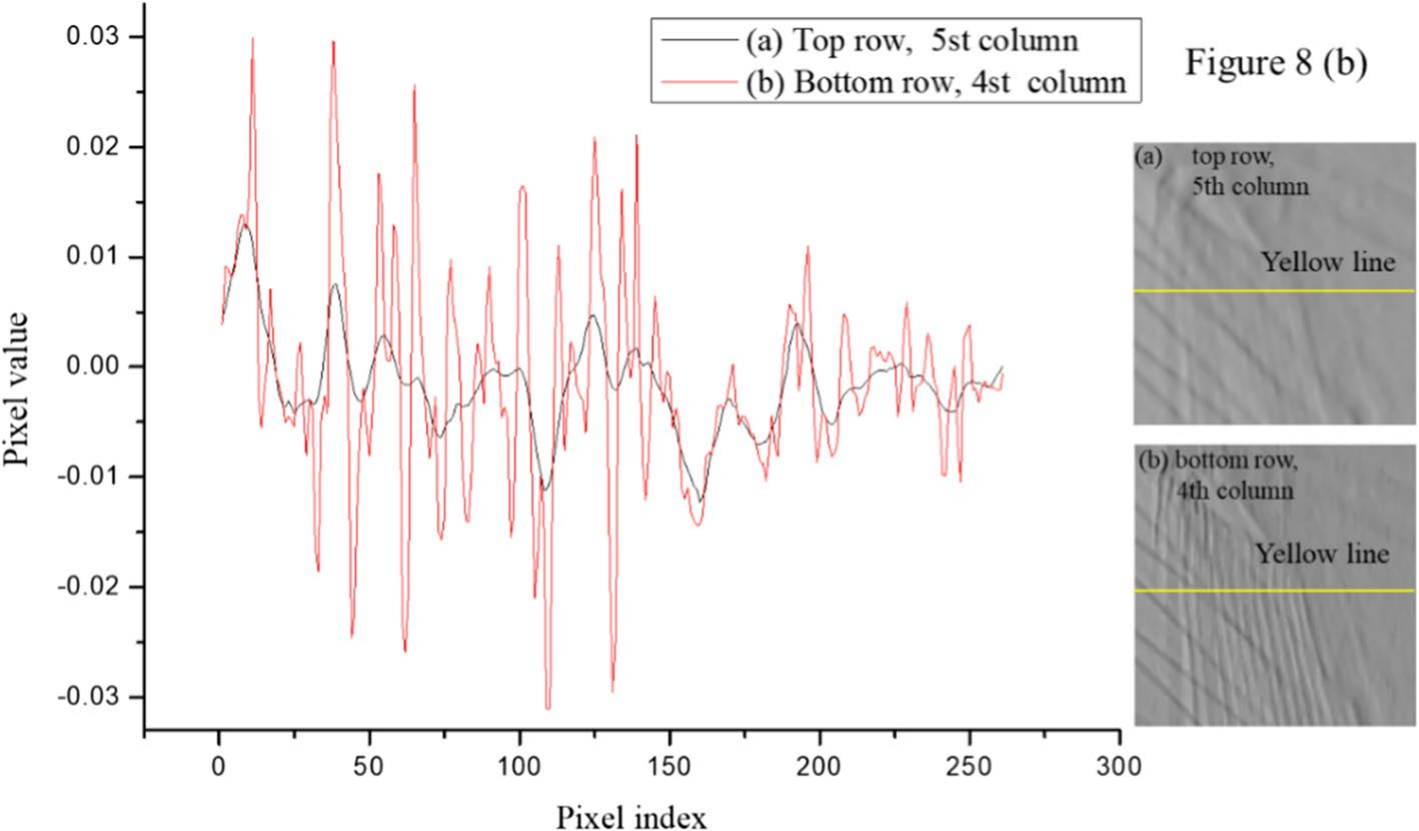
Pixel profiles for the yellow line in the images in the **a** top row, fifth column and **b** bottom row, fourth column in Fig. 8**b**

A comparison of the pixel profiles in Fig. 9 revealed that the noise in the comparison image (simple bitonic filter with a parameter value of 9) was mostly removed, but the detailed information from the sample was severely damaged. In contrast, the pixel profile for the multi-resolution structurally varying bitonic filter with a parameter value of 7 showed that the noise was strongly suppressed, while the detailed information was well preserved. These results verify the strong performance of the proposed filter for the simultaneous preservation of detailed information and noise removal (Best PSNR: 27.54 dB, MSE: 0.0019, and SSIM: 0.99).

**Fig. 10:**
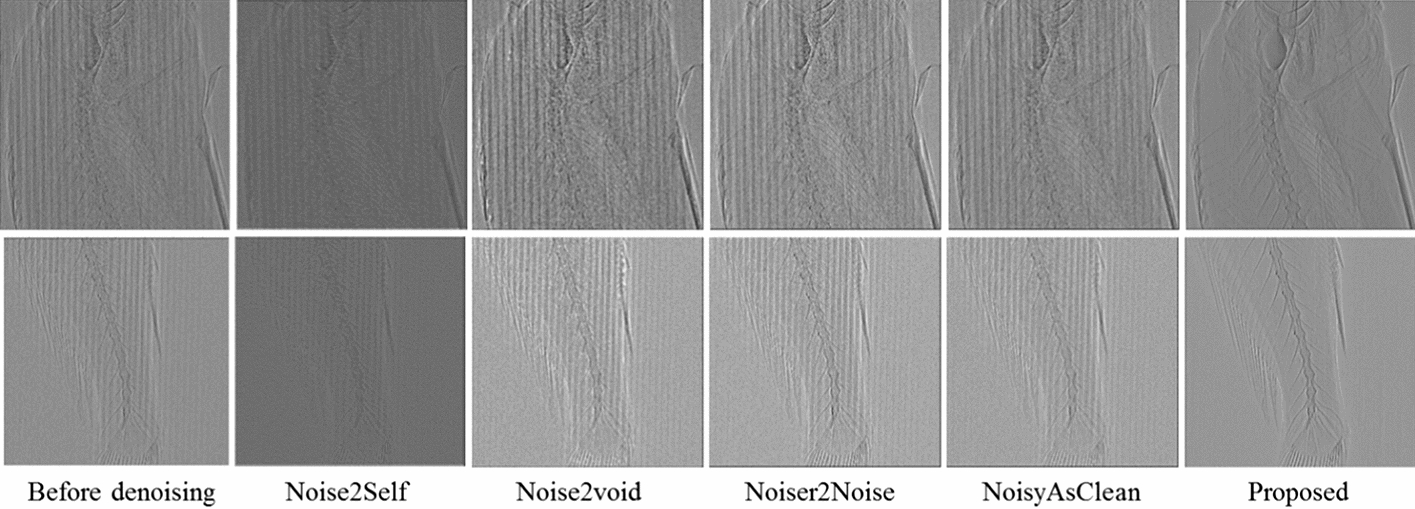
Images of the proposed filter and representative unsupervised deep-learning-based denoising methods Noise2Self, Noise2Void, Noiser2Noise, and NoisyAsClean

In Fig. 10, we compared the images from the proposed filter with those generated by recent deep learning-based denoising methods. Considering that it is generally difficult to obtain a high-quality, pristine reference image for the proposed grid-based phase-contrast X-ray images, the representative unsupervised learning-based denoising methods Noise2Self [46], Noise2Void [47], Noiser2Noise [48], and NoisyAsClean [49] were employed for the performance comparison. Figure 10 presents the images. The proposed filter provided clearer and more detailed inner and outer structures of the biological sample than did the deep learning-based denoising methods. We have not presented the results for the removal of the Moire artifacts using the adaptive Gaussian notch filter because Moire artifacts were not generated in our results. However, Varghese et al. [50] clearly demonstrate the removal of Moire artifacts using an adaptive Gaussian notch filter (Fig. 7 of the cited paper). We successfully removed the periodic noise in a similar manner to the Moire artifacts. The residual noise was also significantly suppressed by the bitonic filters. Thus, we demonstrated excellent noise removal while maintaining the internal information of the biological sample. For reference, we used the CT image dataset in related to the deep learning models [62].

### Comparison of TV-L1 denoising

**Fig. 11:**
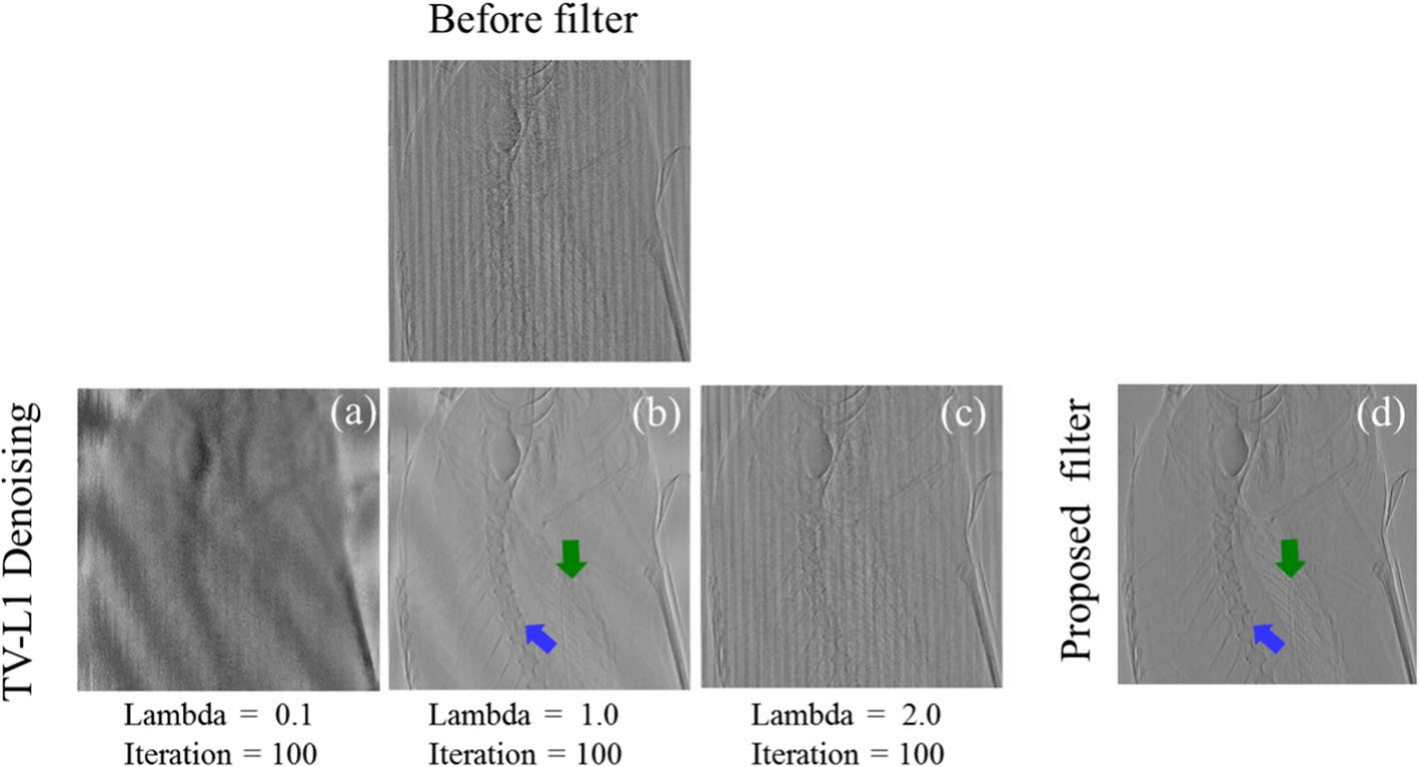
Images produced by the TV-L1 denoising and proposed filters. The top row presents the image before filtering: **a**–**c** images from the TV-L1 denoising filter with a lambda of 0.1, 1.0, and 2.0, respectively, and 100 iterations and **d** image from the proposed filter

Figure 11 presents filtered images produced by adjusting the parameters for the TV-L1 denoising filter [51]. The image produced by TV-L1 denoising filter with a lambda of 1.0 (Fig. 11b) was most comparable with that produced by the proposed filter (Fig. 11d). However, Fig. 11b shows Moire artifacts in the diagonal direction, while Fig. 11d does not. In addition, Fig. 11b vaguely represents the vertebrae (blue arrow) and comb fins (green arrow), while Fig. 11d presents them clearly and in detail. Therefore, the proposed filter had a stronger filtering ability than did the TV-L1 denoising filter.

## Conclusion

In this study, we proposed a method for the production of clear, high-resolution phase-contrast X-ray images using instantaneous frequency and noise filtering. Conventional absorption, scattering, and phase-contrast X-ray images produced using Fourier analysis suffer from ambiguous internal structures and noise patterns. Compared with conventional images, the proposed method generates images that show a clear division between the inner and outer boundaries and detailed internal structures. Moreover, a pixel profile comparison of absorption *x*-axis gradient and the proposed images revealed that the proposed image offers the clear and precise distinction between bone and soft tissue details and reveals detailed structures. Thus, the proposed imaging method holds promise for use in real-time bio-imaging using 3D CT.

## Method and experimental setup

### Data acquisition

**Fig. 12:**
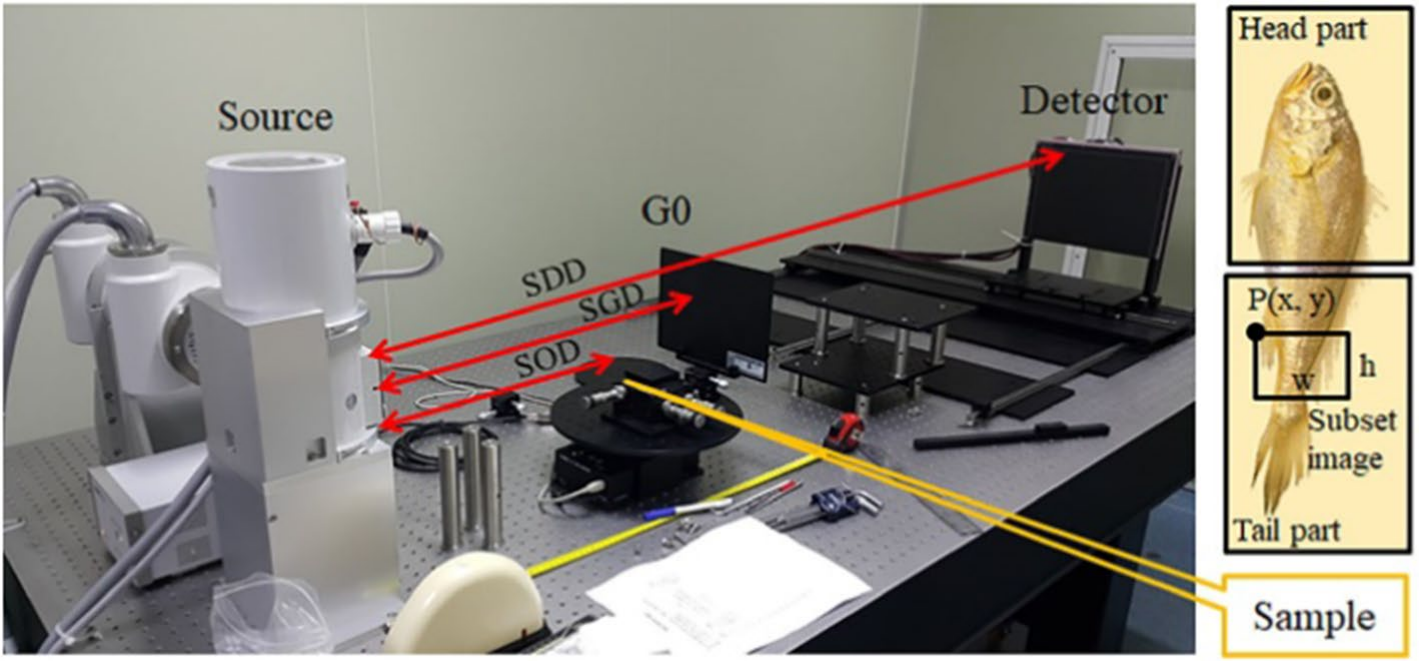
Setup of the equipment used to obtain the raw images. The X-ray tube was operated at 22 kVp and 20 mA, with an exposure time of 630 ms. The focal spot size of the equipment was 100 μm and the grid pitch was 0.118 mm (215 dpi). The CMOS flat-panel detector had a pixel size of 7 µm and dimensions of 3840 × 3072. The source-to-object distance (SOD), source-to-grid distance (SGD), and source-to-detector distance (SDD) were 500, 700, and 1448 mm, respectively

The components of the equipment used to obtain the raw images are presented in Fig. 12. The setup included an X-ray source that was operated at 22 kVp and 20 mA with an exposure time of 630 ms and a focal spot size of 100 μm, a grid with a pitch size of 0.118 mm (215 dpi), and a detector with a pixel size of 7 µm and dimensions of 3840 × 3072. The source-to-sample distance was 500 mm, the source-to-grid distance was 700 mm, and the source-to-detector distance was 1448 mm. A yellow corvina (Larimichthys polyactis) with a maximum thickness of 2 cm was used as the bio-sample. Subset images of the head and tail of the bio-sample were obtained in this study.

### Phase-contrast X-ray image by instantaneous frequency and noise filtering

**Fig. 13:**
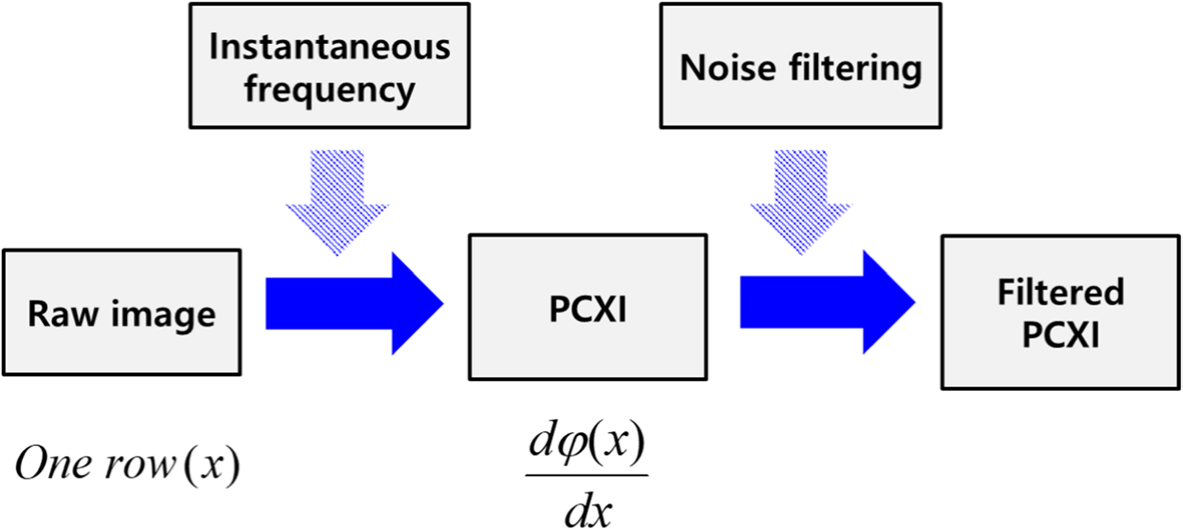
Acquisition of a phase-contrast X-ray image using instantaneous frequency and noise filtering. PCXI: phase-contrast X-ray image

**Fig. 14:**
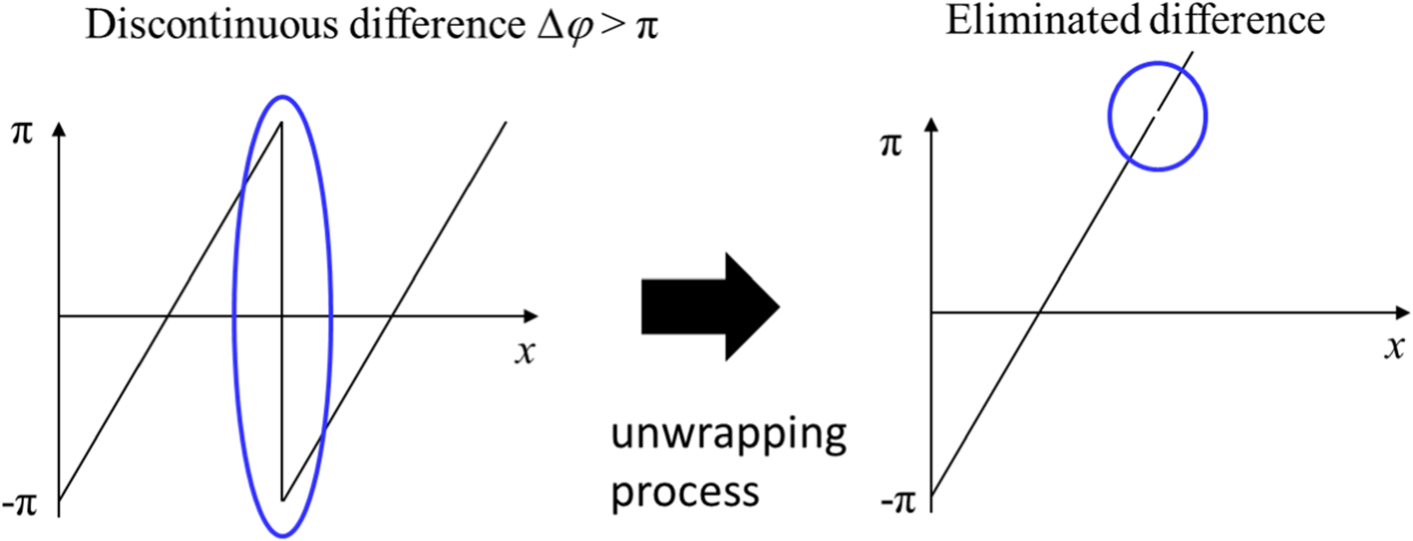
Description of the unwrapping process. ± 2π is added to the phase value at the position of the difference Δ*φ* until the difference is less than π

**Fig. 15:**
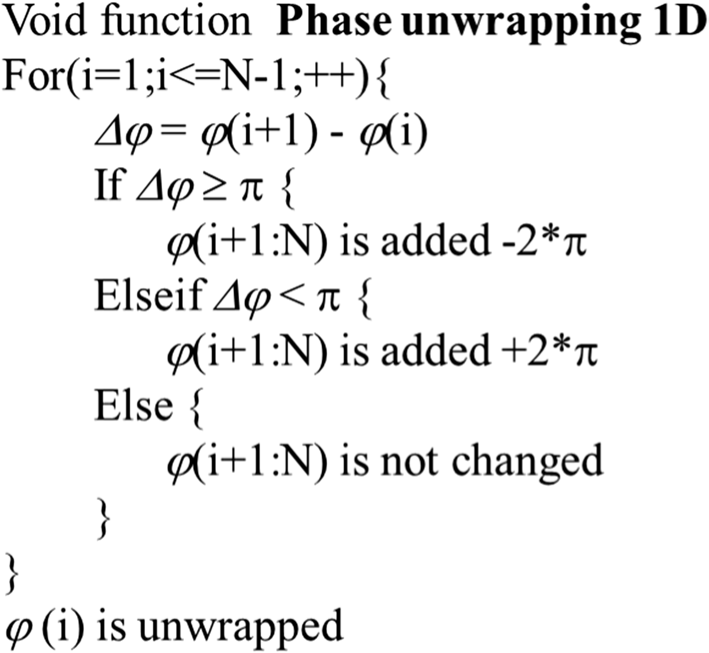
Pseudocode of the unwrapping process. ± 2*π* was added to the phase value at the position of the difference Δ*φ* until the difference was less than *π*. *φ* (*i*) is the phase value at index *i*. *φ* (i + 1:N) are the phase values from indexes i + 1 to N

Figure 13 shows the acquisition process for a phase-contrast X-ray image using instantaneous frequency using One row(*x*) in the raw image. This line was first transformed into a conjugate line Hilbert transform (HT), meaning that One row(x) becomes HT (One row(*x*)). Generally, an HT converts a cos signal into a sin signal or a sin signal into a cos signal. Subsequently, the line and conjugate line were combined to form complex line exp *φ*(*x*) (Eq. 1). The complex line was guided to a phase line *φ*(*x*), after which the phase line was unwrapped (unwrapp* φ*(*x*) in Eq. 2). The unwrapping process in Eq. (2) was conducted using a function in Matlab™ and is described in Fig. 13. During the unwrapping process, if the discontinuous difference between phase values was larger than π, ± 2π was added to the phase value at the position of the difference until the difference was less than π as shown in Fig. 14. Thus, the discontinuous difference between phase values was eliminated [52]. The pseudocode of the unwrapping process is shown in Fig. 15. The gradient d*φ*(*x*)/d*x* of the unwrapped phase line was converted to an instantaneous frequency line in Eq. (3), where *f*_s_ corresponds to the length of a line. In Eqs. (1) and (3), *HT* denotes the Hilbert transform, and *Insfre* is the instantaneous frequency. The gradient d*φ*(*x*)/d*x* is a line from the phase-contrast X-ray image using instantaneous frequency [53].

1 $$ {\text{One}}\;{\text{row}}(x) + HT({\text{One}}\;{\text{row}}(x))i = \exp [\phi (x)], $$

2 $$ \phi (x) \to {\text{unwrapp}}\;(\phi (x)) = \phi_{{{\text{unwrapp}}}} (x), $$

3 $$ \frac{{f_{s} }}{2\pi } \cdot \frac{{{\text{d}}\phi_{{{\text{unwrapp}}}} (x)}}{{{\text{d}}x}} = {\text{Insfre}}(x), $$

### Conventional images by Fourier analysis

**Fig. 16:**
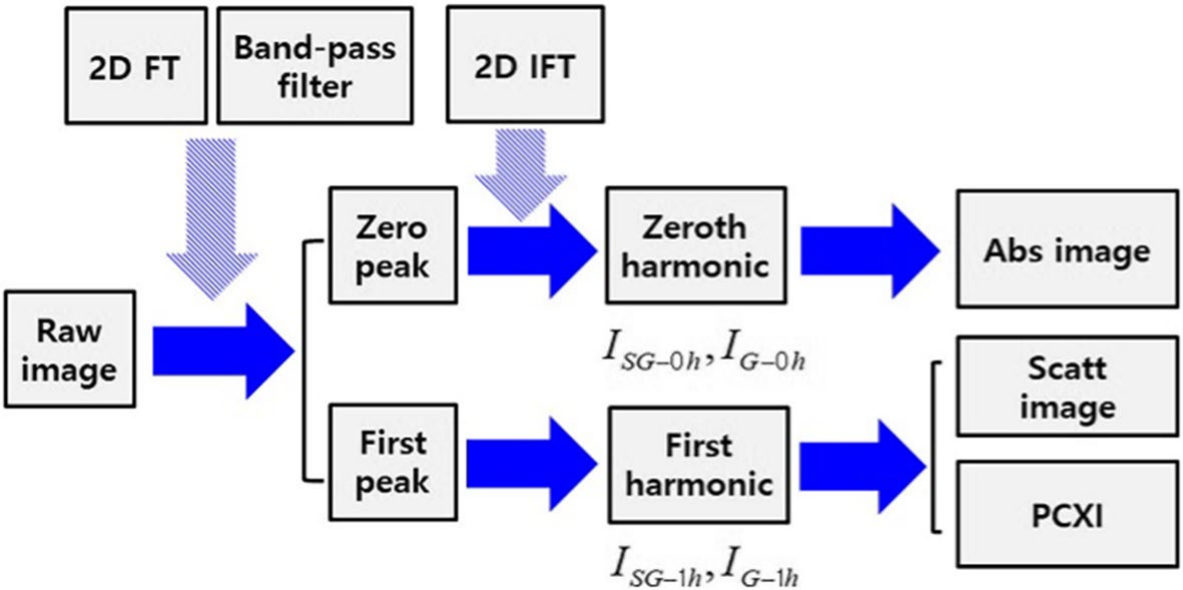
Absorption, scattering, and phase-contrast X-ray images obtained using Fourier analysis. 0 h: zeroth harmonic; 1 h: first harmonic; FT: Fourier transform; IFT: inverse Fourier transform; Abs: absorption image; Scatt: scattering image; PCXI: phase-contrast X-ray image; G: grid; SG: sample + grid

Figure 16 presents a schematics diagram for the simultaneous acquisition of absorption, scattering, and phase-contrast X-ray images from raw images using Fourier analysis. The raw images (grid and sample + grid) obtained by the detector were transformed into Fourier domain images expressed as a complex number. The Fourier domain images were separated into zero and first peak areas using a bandpass filter. The inverse Fourier transform of the zero and first peak areas was the zero and first harmonic images, respectively. The absorption image was obtained by dividing the ratio of the zero harmonic image of the sample + grid by that of the grid. The scattering and phase-contrast X-ray images were obtained by dividing the ratio and angle by the combination of the zero and first harmonic images of the grid and sample + grid. The absorption, scattering, and phase-contrast X-ray images obtained using Fourier analysis were calculated using Eqs. (4)–(7) [54, 55], where *Abs*, *Scatt*, and *PCXI* denote the absorption, scattering, and phase-contrast X-ray images, respectively. *SG* and *G* are the sample + grid and grid, respectively. 0 h and 1 h represent the zero and first harmonic images, respectively. *Scatt-PCXI* is a common formula in scattering and phase-contrast X-ray images. Additionally, the grid and sample + grid images only show the grid and sample with the grid in the detector area, respectively.

4 $$ I_{{{\text{Abs}}}} = \frac{{I_{{{\text{SG}} - 0h}} }}{{I_{G - 0h} }}, $$

5 $$ I_{{{\text{Scatt}} - PCXI}} = \frac{{I_{{{\text{SG}} - 1h}} }}{{I_{G - 1h} }} \cdot \frac{{I_{G - 0h} }}{{I_{SG - 0h} }}, $$

6 $$ I_{{{\text{Scatt}}}} = \left| {I_{{{\text{Scatt}} - PCXI}} } \right|, $$

7 $$ I_{PCXI} = \tan^{ - 1} \left( {\frac{{{\text{Im}} \left( {I_{{{\text{Scatt}} - PCXI}} } \right)}}{{{\text{Re}} \left( {I_{{{\text{Scatt}} - PCXI}} } \right)}}} \right). $$

### Notch adaptive Gaussian filter

**Fig. 17:**
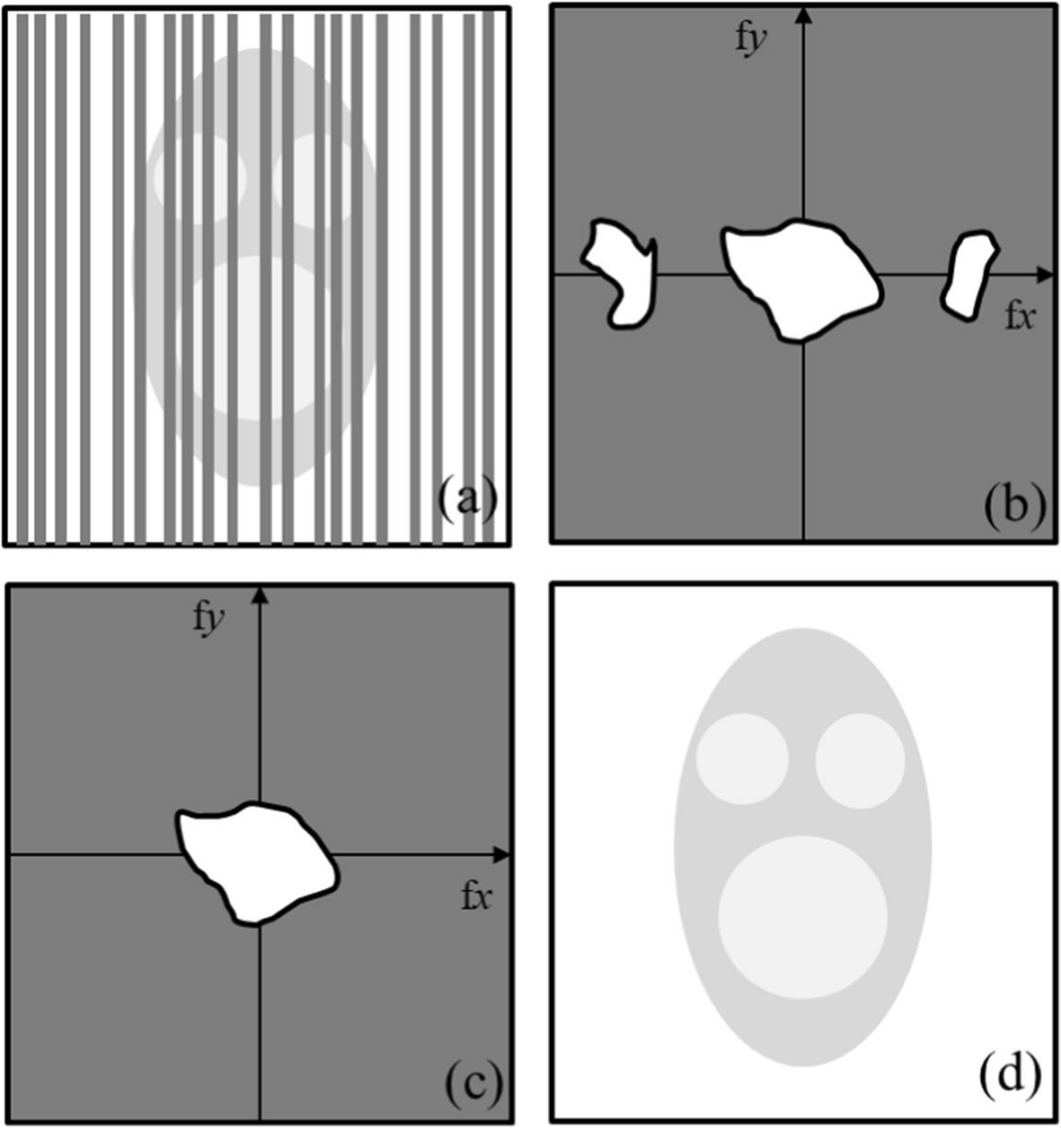
Basic concept of an adaptive Gaussian notch filter: **a** image, **b** Fast Fourier transform (FFT) amplitude distribution, **c** removal of the frequency components of the periodic noise using segmentation and extraction algorithms and FFT amplitude distribution with the frequency components removed, and **d** final filtered image. *f*_*x*_: *x*-axis spatial frequency; *f*_*y*_: *y*-axis spatial frequency

Figure 17 presents a schematic illustration of the operation of the adaptive Gaussian notch filter. Figure 17a displays an image of a face with periodic noise, which was converted using a Fast Fourier transform (FFT) into the image in Fig. 17b. Figure 17b shows the FFT amplitude distribution. The two unwanted surrounding stain patterns (i.e., frequency components of the periodic noise) in Fig. 17b were removed using segmentation and extraction algorithms (adaptive thresholding and region growing, respectively). The removed amplitude distribution is presented in Fig. 17c. Subsequently, the image in Fig. 17c was converted into the image in Fig. 17d using an inverse FFT. Therefore, Fig. 17d represents the desired image for an adaptive Gaussian notch filter.

Segmentation and extraction algorithms in the spatial frequency are essential for the effective performance of an adaptive Gaussian notch filter. This could be attributed to the fact that if a signal is stationary (i.e., if the spatial frequency of the signal is constant in space), a notch filter with a fixed frequency range can be used to easily remove the periodic noise from that signal, whereas this is not the case if the signal is non-stationary (i.e., if the spatial frequency of the signal changes in space). To solve this problem arising from a notch filter with a fixed frequency range, we characterized the noise from the signal, and this noise was adaptively removed by applying a suitable filter for the noise characteristics. The identification of these characteristics and their extraction are achieved using the segmentation and extraction algorithms. An adaptive notch filter can adaptively remove periodic noise from a signal by identifying the notch frequency range corresponding to the location of the filter operation [56]. As such, previous studies have reported the successful use of adaptive notch filters and these algorithms. Moallem et al. [57] used the segmentation and extraction algorithms with an adaptive threshold and region growing, while Chakraborty et al. [58] extracted most of the frequency components from noise using appropriate thresholding and filtration methods (i.e., using an adaptive sinc restoration filter to detect the noise spectrum profile). Alibabaie et al. [59] also successfully eliminated noise from an image using a fuzzy transform.

The notch adaptive Gaussian filter [50] proposed in our study can segment and extract periodic noise by adaptively analyzing the average spatial frequency from a change in an adjacent signal. Consequently, this analysis enables the effective elimination of periodic noise by changing the application size of the notch filter.

### Bitonic filters: the selection of the multi-resolution structurally varying bitonic filter

Bitonic filters fundamentally operate based on a sorting process (i.e., descending or ascending sorting, where there is only one minimum and one maximum in a given region). For example, a median filter ranks the pixel values in a given region, after which the middle-ranked (centile: 50%) value in the sorted list (descending or ascending) is used as the output. The rank in a median filter can be selected using various orders, and it can be used to eliminate impulsive noise while preserving monotonically increasing or decreasing signals [60].

Bitonic filters consist of a morphological filter (for opening and closing operations) and a Gaussian linear filter. The morphological filter transforms the input image into an intermediate result. The operation of the morphological filter is based on that of a rank filter. The closing operation by the morphological filter preserves the signals with local maxima and removes the signals with local minima from the image. In contrast, the opening operation removes the signals with local maxima and preserves the signals with local minima. Consequently, the opening and closing operations of the morphological filter remove most of the noise from the image. Lastly, the intermediate result is converted into a filtered image using the Gaussian linear filter. The Gaussian linear filter removes the residual noise after the morphological filtering [60].

Three types of bitonic filter were employed in the present study: a simple bitonic filter, a structurally varying bitonic filter, and a multi-resolution structurally varying bitonic filter. The simple bitonic filter used the morphological filter and Gaussian linear filter described above, and the image pixels within the circular mask area were used for morphological filtering. The morphological filter and Gaussian linear filter were also employed in the structurally varying bitonic filter. However, in contrast to the simple bitonic filter, the structurally varying bitonic filter employed various mask shapes for morphological filtering, rather than just circular. The shape of the mask was selected based on the information in the image pattern in the local area. Particularly, the information in the image pattern consists of a matrix of the first and second differential gradients of the image pixels on the x and y axes in the local area. This matrix determined the shape (ellipse) and orientation (slope of the ellipse) of the mask. A structurally varying bitonic filter can thus adaptively apply various mask shapes using the matrix. Therefore, it represents an adaptive filter based on the surrounding information.

The multi-resolution structurally varying bitonic filter transforms an input image into a filtered image (*I*_F_: *I* → Filter). In addition, this filter can reduce (*I*_FR_: *I* → Filter → Reduction) and enlarge (*I*_FRE_: *I* → Filter → Reduction → Enlargement) the filtered image (*I*_F_). The state of the image before the reduction is referred to as the upper level and the state of the image after reduction is referred to as the lower level. In the present study, the reduced image (*I*_FR_) was subjected to low-level calculation (*I*_FR → LLC_: *I* → Filter → Reduction → Low-Level Calculation), after which the reduced image (*I*_FR_) was enlarged (*I*_FRE_: *I* → Filter → Reduction → Enlargement). The upper level was calculated using *I*_F_ − *I*_FRE_ + *I*_FR → LLC_, and the lower level was calculated using the same method used to calculate the upper level. The multi-resolution structurally varying bitonic filter employs a multi-level recursion calculation structure. Therefore, this filter can be used to obtain a final image via the combined operation of image reduction and enlargement [31].

The structurally varying and multi-resolution structurally varying bitonic filters simultaneously achieved the preservation of discontinuities (smooth or edges) in the signal and noise removal. In addition, they exhibited robust performance in terms of noise removal at various noise levels [61]. In this study, we selected the multi-resolution structurally varying bitonic filter for our proposed method.

### Training details for the unsupervised deep-learning-based denoising algorithms

We tested the denoising performance of the unsupervised learning methods Noise2Self [46], Noise2Void [47], Noiser2Noise [48], and NoisyAsClean [49]. These networks were implemented using the PyTorch library. To prevent the overfitting of the models due to insufficient data, we derived a pre-trained model from a large Mayo benchmark dataset of grayscale CT images [62] with similar characteristics to our grayscale data and then fine-tuned our data. We trained the comparison models for up to 200 epochs and the mean squared error loss function was minimized using the Adam optimizer. The learning rate was set to 0.0001, and the batch size was set to 32.

## Data Availability

All data generated or analyzed during this study are included in the article.

## Abbreviations

PSNR: Peak signal-to-noise ratio
CT: Computed tomography
3D: Three dimensional
Abs: Absorption image
Scatt: Scattering image
PCXI: Contrast X-ray image
SG: Sample + grid
G: Grid
0 h: Zero harmonic image
1 h: First harmonic image
Scatt-PCXI: A common formula in scattering and phase-contrast X-ray image
Grid image: Only a grid image in the detector area
Sample + grid image: A sample image with the grid in the detector area
HT: Hilbert transform
unwrapp: Unwrapped
Insfre: Instantaneous frequency
FFT: Fast Fourier transform
